# Induced TRIM21 ISGylation by IFN-*β* enhances p62 ubiquitination to prevent its autophagosome targeting

**DOI:** 10.1038/s41419-021-03989-x

**Published:** 2021-07-13

**Authors:** Jie Jin, Xianbin Meng, Yi Huo, Haiteng Deng

**Affiliations:** 1 MOE key Laboratory of Bioinformatics, Center for Synthetic and Systematic Biology, School of Life Sciences, Tsinghua University, Beijing, China; 2 BeiGene (Beijing) Co., Ltd., Beijing, China

**Keywords:** Proteins, Autophagy, Innate immunity, Post-translational modifications

## Abstract

The tripartite motif-containing protein 21 (TRIM21) plays important roles in autophagy and innate immunity. Here, we found that HECT and RLD domain containing E3 ubiquitin protein ligase 5 (HERC5), as an interferon-stimulated gene 15 (ISG15) E3 ligase, catalyzes the ISGylation of TRIM21 at the Lys260 and Lys279 residues. Moreover, IFN-*β* also induces TRIM21 ISGylation at multiple lysine residues, thereby enhancing its E3 ligase activity for K63-linkage-specific ubiquitination and resulting in increased levels of TRIM21 and p62 K63-linked ubiquitination. The K63-linked ubiquitination of p62 at Lys7 prevents its self-oligomerization and targeting to the autophagosome. Taken together, our study suggests that the ISGylation of TRIM21 plays a vital role in regulating self-oligomerization and localization of p62 in the autophagy induced by IFN-*β*.

## Introduction

Type I interferons (IFNs) are ubiquitously expressed in host cells upon stimulation by pathogen-associated molecular patterns, such as nucleic acids and lipopolysaccharide, derived from viruses and bacteria. They are known to play versatile roles in the innate and acquired immune responses partially by inducing the expression of a series of downstream genes, known as interferon-stimulated genes (ISGs) to eliminate exogenous pathogens from the host body [1].

Interferon-stimulated gene 15 (ISG15), a highly inducible ISG, was first identified by Haas et al. [2] as a ubiquitin-like protein expressing a 17-kDa precursor protein comprising 165 amino acid residues. The phenomenon of ISGylation involves the proteolytic processing of the C-terminus of ISG15 to expose the LRLRGG hexapeptide motif prior to the conjugation of the same to its target proteins at specific lysine residues [3]. Similar to ubiquitination, ISGylation is a reversible process catalyzed by its specific E1 activating enzyme (ubiquitin-activating enzyme E1-like; UBE1L [4]), E2 conjugating enzyme (ubiquitin-conjugating enzyme H8; UBCH8 [5]), and E3 ligases [HECT and RLD domain containing E3 ubiquitin protein ligase 5 (HERC5) [6, 7], estrogen-responsive finger protein (EFP) [8], or human homolog of Ariadne (HHARI) [9]] in a stepwise manner, while the deISGylation from its targets is mediated by ubiquitin-specific peptidase 18 (USP18) [10] reversibly. A direct relationship between ISGs and autophagy has been confirmed in a recently published study, wherein ISGylation of BECN1 induced by type I IFNs was demonstrated to be critical in regulating autophagy by competing for its K63-linked ubiquitination [11]. Inspired by this vital mechanistic insight, we aimed to identify other ISGylated proteins involved in the regulation of autophagy. Although hundreds of cellular proteins were documented as being ISGylated in two proteomic profiling studies [6, 12], the results from these studies still remain to be validated, and little is known regarding the functional regulation of these ISGylated proteins.

In the present study, we verified tripartite motif-containing protein 21 (TRIM21) as a substrate of ISGylation, which was also detected in a recent publication [6]. TRIM21 is a dual function protein that functions not only as a cytosolic Fc receptor [13, 14] sensing intracellular incoming antibody-bound viruses to initiate immune responses [15, 16], but also as an E3 ubiquitin ligase catalyzing self-ubiquitination [17] and the ubiquitination of substrates with different linkages. For instance, TRIM21 catalyzes the mono-ubiquitination of IKK*β* [18], K27-linked ubiquitination of MAVS [19], and K63-linked ubiquitination of Nmi [20]. p62 functions as a selective autophagy receptor responsible for selecting ubiquitin-tagged cytoplasmic components destined for lysosomal degradation. It undergoes self-oligomerization and co-aggregation with ubiquitinated substrates for autophagic cargo segregation, which is precisely regulated by post-translational modifications [21]. Coincidentally, the self-oligomerization of p62, a prerequisite for its localization to the autophagosome [22], is blocked by the TRIM21-mediated K63-linked ubiquitination of its Lys7 site [23]. Autophagy, the highly conserved intracellular pathway for recycling damaged proteins and organelles, also participates in innate and acquired immune responses, including the elimination of intracellular pathogens, inflammation control, and MHC-restricted antigen presentation during viral and bacterial infection [24, 25]. Consistently, autophagy is assumed to be activated by the nutrient-sensing pathways because of the consumption of intracellular nutrients by invading pathogens, and it is integrated with pattern recognition receptor-mediated immune-sensing pathways [24]. Furthermore, it has been recently reported that autophagy can be directly induced by type I IFNs in different cells [26–28]. However, the role of type I IFN signaling, especially ISGs, in the induction and feedback regulation of autophagy has not yet been comprehensively investigated [29]. We found that the ISGylation of TRIM21 induced by IFN-*β* elevates the levels of K63-linked ubiquitination of TRIM21 as well as p62 by enhancing its E3 ubiquitin ligase enzymatic activity specific for K63-linkage, preventing p62 oligomerization and subsequent localization to the autophagosome.

## Results

### TRIM21 is ISGylated by HERC5

**Fig. 1:**
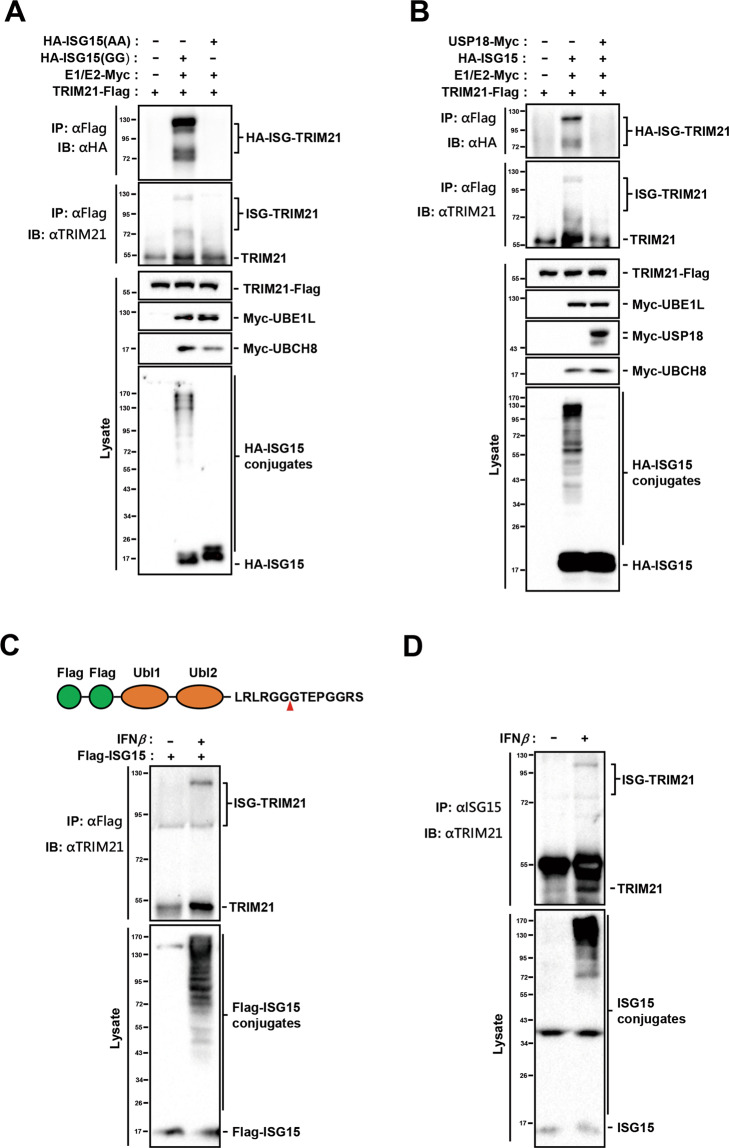
TRIM21 can be ISGylated in HEK293 and A549 cells. **A** TRIM21-Flag, UBE1L-Myc (E1), and UBCH8-Myc (E2) were overexpressed together with HA-ISG15(GG) or HA-ISG15(AA) in HEK293 cells. Cells were collected and lysed 24 h after transfection, and cell lysates were incubated with mouse Flag antibodies and protein A/G agarose at 4 °C for 4 h or overnight, followed by western blot with HA and TRIM21 antibodies. Cell lysates were also detected with Flag, Myc, and HA antibodies. **B** TRIM21-Flag, UBE1L-Myc (E1), UBCH8-Myc (E2), and HA-ISG15 were co-expressed with or without USP18-Myc in HEK293 cells. Cell lysates were subjected to immunoprecipitation with mouse Flag antibodies. The immunoprecipitates and the lysates were analyzed with TRIM21, Flag, Myc, and HA antibodies. **C** Flag-ISG15 overexpressing A549 cells were treated with 2000 unit/ml IFN-*β* for 48 h. ISGylated proteins were pulled down by mouse Flag antibodies and detected by TRIM21 antibodies. Cell lysates were subjected to western blot with Flag antibodies. **D** A549 cells treated with 2000 unit/ml IFN-*β* for 48 h were lysed and immunoprecipitated with ISG15 antibodies, followed by immunoblotting with TRIM21 antibodies. Cell lysates were analyzed with ISG15 antibodies. Data shown are representative of three independent experiments.

To identify new substrates of ISGylation, we cloned all genes constituting the ISG15-conjugating system, including ISG15, UBE1L, UBCH8, HERC5, EFP, HHARI, and a subset of potential target genes reported previously [6, 12]. These potential target genes were then respectively co-transfected with ISG15, UBE1L, and UBCH8 in human embryonic kidney 293 (HEK293) cells. Among them, PKM2 and TRIM21 were found to be ISGylated in immunoprecipitation and immunoblotting analyses (Fig. S1), in which only PKM2 was validated [6]. In the present study, we characterized the ISGylation of TRIM21 and investigated its functions. Transfection of HEK293 cells with wild-type (ISG15-GG), but not the mutant ISG15 (ISG15-AA), revealed the presence of two ISGylated TRIM21 bands (Figs. 1A and S1). However, the ISGylated bands were undetectable when the deISGylation enzyme USP18 was introduced into the system (Fig. 1B). To confirm whether TRIM21 is also ISGylated under physiological conditions, we treated human non-small-cell lung carcinoma A549 cells with IFN-*β* or IFNA2 and found that the expression of all the studied components of the ISG15-conjugating system were highly elevated, and the two ISGylated bands of TRIM21 were evident (Figs. 1C, D and S2). Taken together, our results demonstrated that TRIM21 is a substrate of ISGylation. The lower ISGylated TRIM21 band was also observed in untreated A549 cells (Figs. 1C, D and S2), suggesting that TRIM21 ISGylation might play other unknown biological roles in the resting state without exogenous IFN-*β* stimulation.

**Fig. 2:**
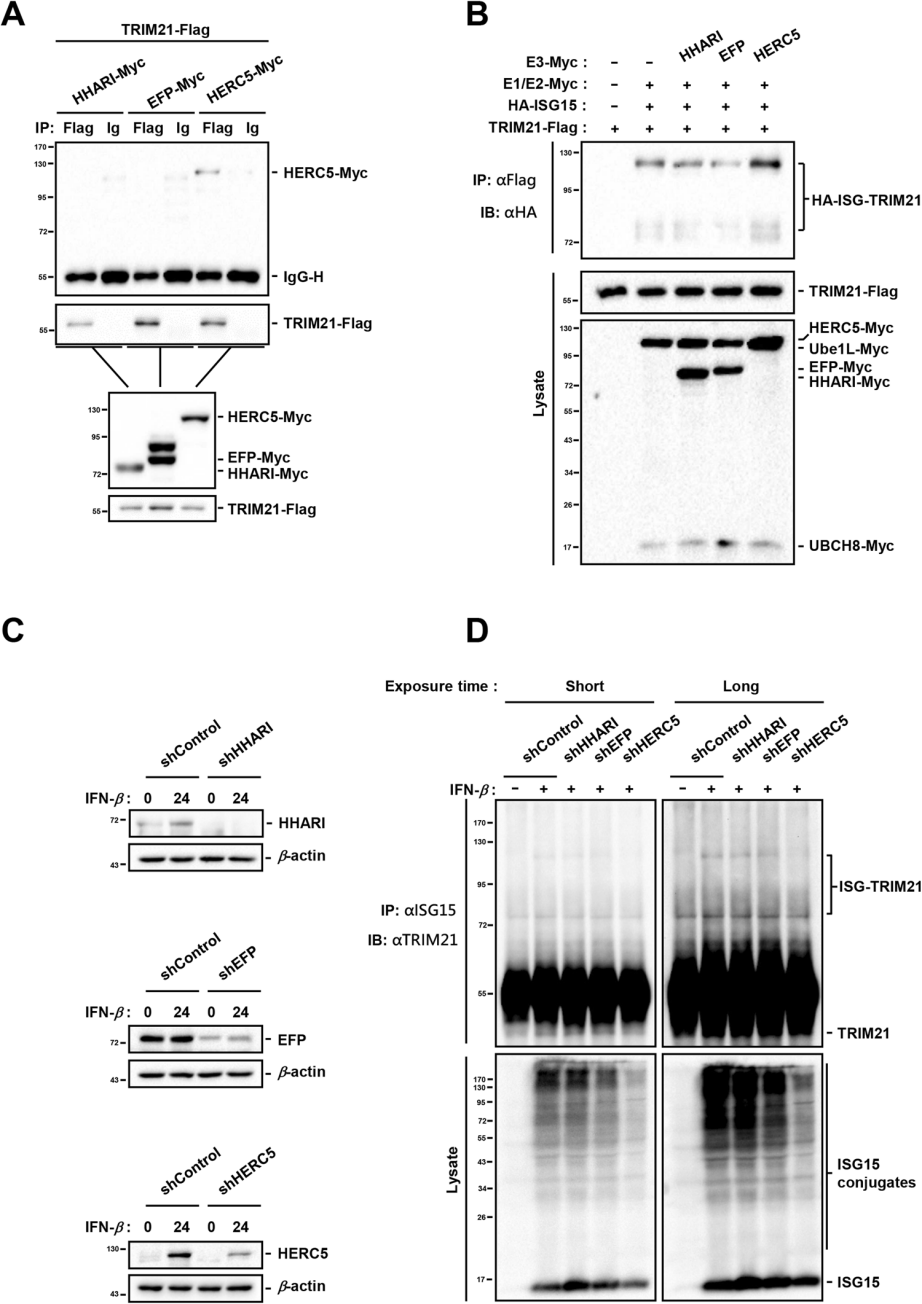
HERC5 catalyzes the ISGylation of TRIM21. **A** TRIM21-Flag was co-expressed with HHARI-Myc, EFP-Myc, or HERC5-Myc in HEK293 cells respectively. Cell lysates were immunoprecipitated with mouse control IgG or mouse Flag antibodies, and the immunoprecipitates were subjected to western blot with rabbit Flag antibodies and mouse Myc antibodies. Cell lysates were also subject to western blot to detect target proteins. **B** TRIM21-Flag, as well as UBE1L-Myc (E1), UBCH8-Myc (E2), and HA-ISG15 were co-transfected with HHARI-Myc, EFP-Myc, or HERC5-Myc into HEK293 cells. Cell lysates were subjected to immunoprecipitation with mouse Flag antibodies, followed by immunoblotting with HA antibodies to detect the ISGylated TRIM21 bands. Cell lysates were analyzed with Myc antibodies. **C**, **D** A549 cells stably transfecting with shControl, shHHARI, shEFP, and shHERC5 were treated with or without IFN-*β* for 24 h. Cells were collected and lysed with RIPA buffer and subjected to immunoblotting with indicated antibodies (**C**), or immunoprecipitation with ISG15 antibodies, followed by immunoblotting with TRIM21 antibodies (**D**). Data shown are representative of three independent experiments.

Among the ISG15-conjugating systems reported, there is only one E1 activating enzyme (UBE1L [4]) and E2 conjugating enzyme (UBCH8 [5]), but there are three E3 ligases (HERC5 [6, 7], EFP [8], or HHARI [9]). To determine which E3 ligase catalyzes the ISGylation of TRIM21, we carried out co-immunoprecipitation analysis, which showed that only HERC5 interacted with TRIM21 (Fig. 2A). More importantly, overexpression of HERC5, but not the other two E3 ISG15 ligases, enhanced the ISGylation of TRIM21 (Fig. 2B). Additionally, endogenous HHARI, EFP, or HERC5 was silenced with the corresponding short hairpin RNA (shRNA) in A549 cells respectively (Fig. 2C). Silencing of HERC5, but not HHARI or EFP, inhibited the TRIM21 ISGylation presented in the top band in IFN-*β*-treated A549 cells (Fig. 2D). These data collectively demonstrate that HERC5 is an E3 ISG15 ligase for the ISGylation of TRIM21.

### Identification of Lys260 and Lys279 in TRIM21 as the major ISGylation sites

**Fig. 3:**
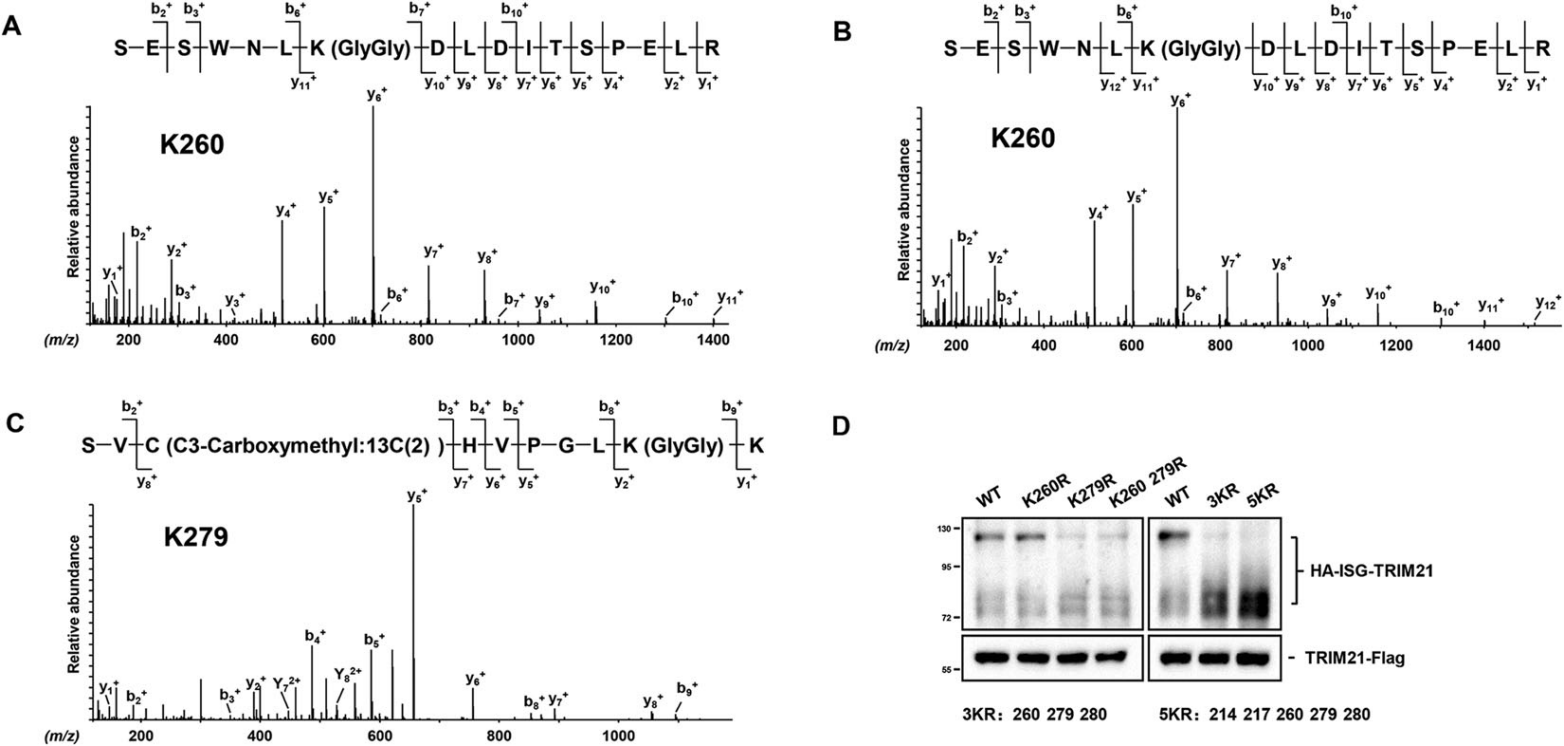
Identification of ISGylation sites in TRIM21. **A**–**C** TRIM21-Flag, as well as the ISG15-conjugating system were overexpressed in HEK293 cells. Cell lysates were pulled down with anti-Flag affinity gel, and then subjected to SDS-PAGE separation and in-gel digestion. Iodoacetic acid-^13^C_2_, instead of iodoacetamide, was used in in-gel digestion to avoid false positive result. LC-MS/MS analysis identified Lys260 as original ISGylation sites in the bottom ISGylated TRIM21 band (**A**), and Lys260, Lys279 as original ISGylation sites in the top ISGylated TRIM21 band (**B**, **C**). **D** HEK293 cells were transfected for 24 h with ISG15-conjuating system and TRIM21 or indicated TRIM21 mutants, K260R, K279R, 2KR (K260/279R), 3KR (K260/279/280R), and 5KR (K214/217/260/279/280R). Cell lysates were subjected to anti-Flag pull-down, followed by immunoblotting with anti-HA and anti-Flag antibody. Data shown are representative of three independent experiments.

Unlike ubiquitin, only one ISG15 molecule is conjugated to the lysine residue in the target proteins [30]. We identified two ISGylated bands of TRIM21 from HEK293 cells in which the ISG15-conjugating system was overexpressed (Fig. 1A, B) or from IFN-*β*-treated A549 cells (Fig. 1C, D). These findings allowed us to hypothesize that there were at least two ISGylation sites in TRIM21. To determine the ISGylation sites in TRIM21, we performed LC-MS/MS analysis. Briefly, the TRIM21-Flag and components of the ISG15-conjugating system were co-overexpressed in HEK293 cells and TRIM21-Flag proteins were immunoprecipitated with anti-Flag affinity gel and then separated by SDS-PAGE, followed by in-gel digestion with trypsin and LC-MS/MS analysis. Our analysis revealed that TRIM21 was ISGylated at the Lys260 site (Fig. 3A) in the bottom band and at both Lys260 and Lys279 in the top band (Fig. 3B, C).

To confirm that Lys260 and Lys279 truly serve as functional ISGylation sites, we mutated these two Lys (K) residues to Arg (R) residues and found that those two ISGylated bands were still visible (Fig. 3D). Moreover, as expected, the top band was evidently lighter in the TRIM21(2KR) mutant than in the wild-type TRIM21 (Fig. 3D). Considering that neighboring Lys residues can be alternatively ISGylated [11, 30], we generated a quintuple mutant TRIM21(5KR) by mutating the additional three lysine residues Lys214, Lys217, and Lys280 in addition to Lys260 and Lys279. The ISGylation assay indicated that the top band, but not the bottom band, disappeared in the TRIM21(5KR) mutant (Fig. 3D). To identify other possible ISGylation sites, the bottom band of the ISGylated TRIM21(5KR) mutant was subjected to in-gel digestion and LC-MS/MS analysis. Subsequent data interpretation identified nine additional ISGylation sites (Fig. S3A–I). However, mutation of all nine Lys residues in TRIM21(5KR) prohibited the protein expression (data not shown). Taken together, our data indicate that Lys260 and Lys279 residues serve as the major ISGylation sites in TRIM21 and that mutation of these residues results in ISGylation on other Lys residues. Considering that the main inducible ISGylation band of TRIM21 by IFN-*β* was the top band (Fig. 1C, D), we chose the TRIM21(5KR) mutant for subsequent functional experiments.

### ISGylation upregulates the K63-linkage-specific E3 ligase activity of TRIM21

**Fig. 4:**
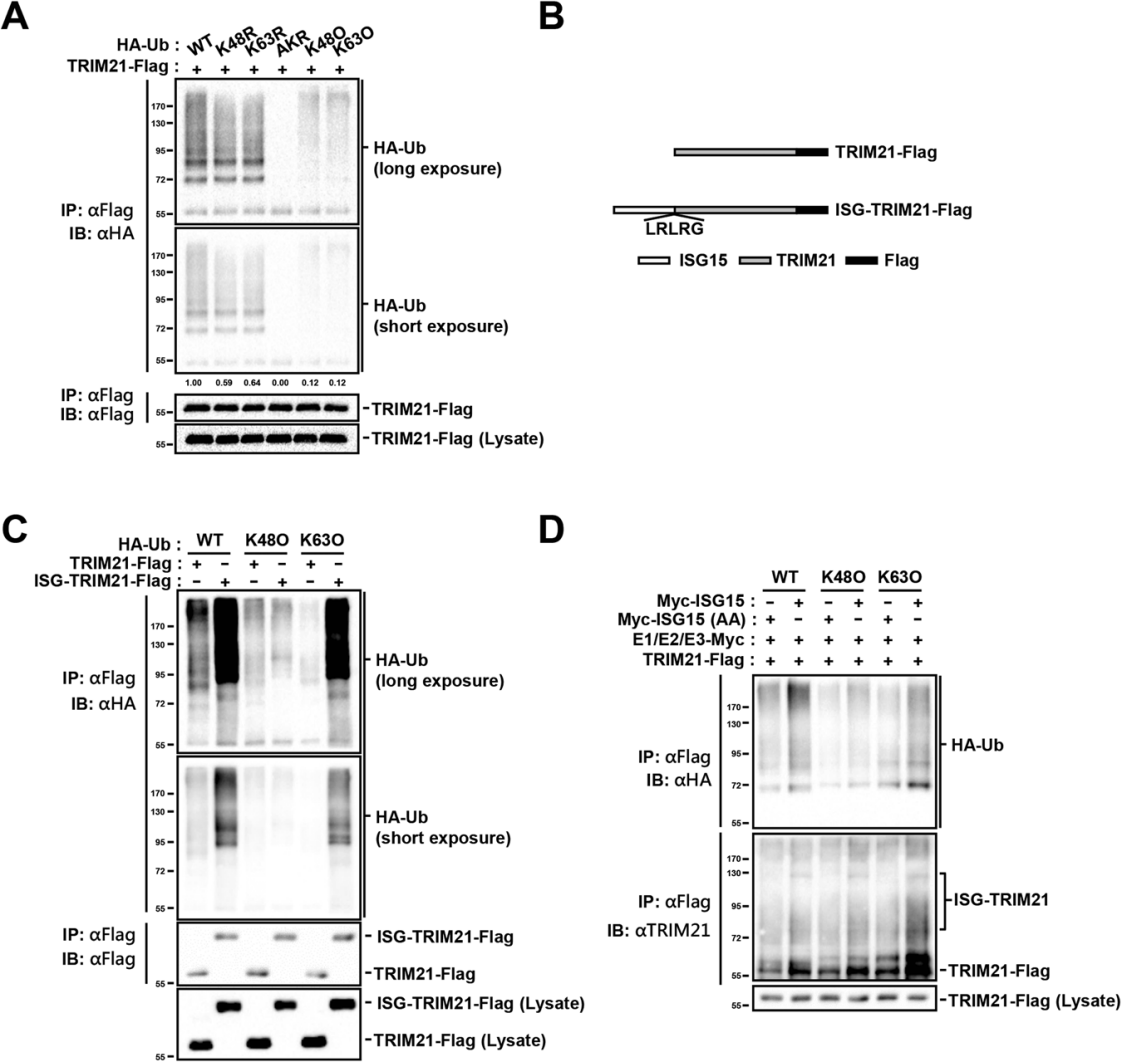
ISGylation increases TRIM21 K63-linked autoubiquitination. **A** TRIM21-Flag was co-expressed with HA-ubiquitin (WT) or HA-ubiquitin mutants (K48R, K63R, AKR, K48O, or K63O) in HEK293 cells. Cell lysates were lysed in denaturing RIPA buffer, followed by immunoprecipitation with mouse Flag antibodies. The immunoprecipitates and cell lysates were detected with rabbit Flag antibodies and HA antibodies. **B** Schematic representation of TRIM21-Flag and ISG-TRIM21-Flag. ISG15-LRLRG was added to the N-terminal of TRIM21 to mimic the ISGylation of TRIM21. **C** TRIM21-Flag or ISG-TRIM21-Flag was co-transfected with HA-unbiquitin (WT) or HA-ubiquitin mutants (K48O or K63O) into HEK293T cells. Cell lysates were pre-processed in denaturing condition as did in **A** and immunoprecipitated with mouse Flag antibodies, followed by immunoblotting with rabbit Flag and HA antibodies. Cells lysates were detected by rabbit Flag antibodies. **D** TRIM21-Flag along with HA-unbiquitin (WT) or HA-ubiquitin mutants (K48O or K63O) were co-expressed with or without ISG15-conjugating system (UBE1L-Myc (E1), UBCH8-Myc (E2), HERC5-Myc (E3), and HA-ISG15) in HEK293 cells. Cell lysates were also denatured as in **A**, and pulled down with mouse Flag antibodies. Cell lysates and immunoprecipitates were detected with rabbit Flag and HA antibodies. Data shown are representative of three independent experiments.

TRIM21 belongs to the RING finger domain containing E3 ubiquitin ligase family and cooperates with numerous E2 ubiquitin-conjugating enzymes, such as UbcH5B, Ube2W, and Ube2N/Ube2V2 heterodimer, to modify itself with mono-ubiquitination and K48- or K63-linked ubiquitination sequentially [17, 31]. To validate these results, we co-transfected Flag-tagged TRIM21 with HA-tagged ubiquitin (WT) or ubiquitin mutants, denoted as K48R in which Lys48 was mutated to Arg, K63R in which Lys63 was mutated to Arg, AKR in which all lysine residues were mutated to arginine, K48O (K48 Only) in which all lysine residues except Lys48 were mutated to arginine, and K63O (K63 Only) in which all lysine residues except Lys63 were mutated to arginine in HEK293 cells, followed by a ubiquitination assay. The results showed that self-ubiquitination of TRIM21 was weaker in the K48R and K63R groups than that in the WT group, and stronger in the K48O and K63O groups than that in the AKR group (Fig. 4A). The findings strongly suggest that TRIM21 catalyzes K48- and K63-linked self-ubiquitination.

Previous reports suggested that ISGylation affects the biological function of targets by competing for K63-linked ubiquitination (BECN1 [11]), or enhancing their E3 ubiquitin ligase enzyme activity (CHIP [32] and parkin [33]). To investigate whether ISGylation increases or reduces the enzymatic activity of TRIM21, we constructed an artificial ISG15-fused TRIM21 by adding an ISG15 mutant with a Gly (G) deletion in the LRLRGG hexapeptide motif to the N-terminus of TRIM21 (Fig. 4B) as previously reported [11, 32]. Interestingly, this ISG15-fused TRIM21, simulating the ISGylation of TRIM21, significantly increased K63-linked, but not K48-linked, self-ubiquitination of TRIM21 (Fig. 4C) in the co-transfection and ubiquitination assays. Additionally, co-expression of the ISG15-conjugating system with TRIM21, which led to the ISGylation of TRIM21, also increased K63-linked ubiquitination of TRIM21 (Fig. 4D). These data collaboratively demonstrate that ISGylation of TRIM21 increases the K63-linkage-specific E3 ligase activity of TRIM21.

### IFN-*β*-induced TRIM21 ISGylation promotes K63-linked ubiquitination of p62

Given that ISGylation stimulates K63-linkage-specific E3 ligase activity of TRIM21, we next investigated whether ISGylation affects the K63-linked ubiquitination levels of other substrates of TRIM21. Three proteins, Nmi [20], Keratin17 [34], and p62 [23], have been documented to be modified by TRIM21-mediated K63-linked ubiquitination under different physiological conditions. Among them, p62 is the only protein that participates in autophagy, a physiological process that was recently reported to be induced by type I IFNs [26–28]. Inspired by the mechanistic studies of ISGylation of BECN1 [11] and CHIP [32], we hypothesized that ISGylation of TRIM21 regulates the autophagy pathway by promoting the K63-linked ubiquitination of p62.

**Fig. 5:**
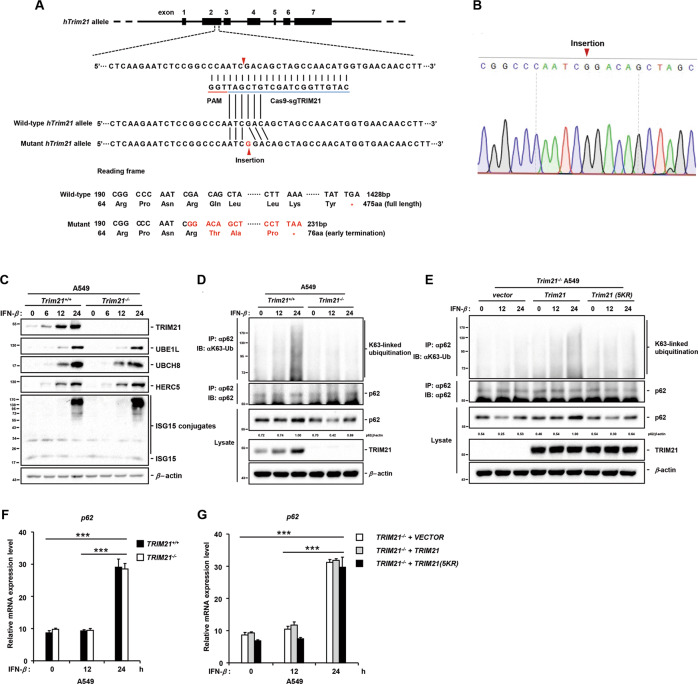
IFN-β-induced TRIM21 ISGylation enhances K63-linked ubiquitination of p62. **A**, **B** Schematic illustration of CRISPR/Cas9-mediated TRIM21 knockout strategy (**A**), and the inserted guanine (G) deoxyribonucleotide into TRIM21 genome was confirmed by sanger sequencing (**B**). **C** *TRIM21*^*+/+*^ and *TRIM21*^*−/−*^ A549 cells were treated with IFN-*β* for indicated timepoints. Cell lysates were lysed in RIPA buffer and then subjected to western blot analysis to detect the protein levels of TRIM21, UBE1L, UCBH8, HERC5, ISG15, and ISG15 conjugates. **D** Lysates of *TRIM21*^*+/+*^ and *TRIM21*^*−/−*^ A549 cells treated with IFN-*β* for indicated timepoints were immunoprecipitated in denaturing RIPA buffer with anti-p62 antibody at 4 °C overnight, followed by immunoblotting analysis to detect indicated proteins. **E** Lysates of IFN-*β*-treated *TRIM21*^*-/-*^ A549 cells stably expressing VECTOR, TRIM21, or TRIM21(5KR) mutant were immunoprecipitated in denaturing RIPA buffer with anti-p62 antibody at 4 °C overnight, followed by immunoblotting analysis to detect indicated proteins. F-G p62 transcripts of IFN-*β*-treated *TRIM21*^*+/+*^ and *TRIM21*^*−/−*^ A549 cells (**F**) or *TRIM21*^*−/−*^ A549 cells stably expressing VECTOR, TRIM21, or TRIM21(5KR) mutant (**G**) were measured by qRT-PCR. Data were analyzed with Two-way ANOVA, and represented as mean ± standard error of the mean (n.s. not significant. ***P < 0.001). Data shown are representative of three independent experiments.

To investigate the proposed hypothesis, we generated a *TRIM21*^*−/−*^ A549 cell line using CRISPR/Cas9 technology with a sgRNA targeting the second exon of the *TRIM21* genome sequence (Fig. 5A). The sequencing data indicated that a single guanine (G) deoxyribonucleotide residue was inserted into the *TRIM21* genome sequence, resulting in a 76aa TRIM21 mutant due to early translation termination of *TRIM21* mRNA caused by frameshift mutation (Fig. 5A, B). The 76aa TRIM21 mutant was not detectable in the immunoblotting assay (Fig. S4). In addition, we detected the protein levels of ISG15-conjugating system members and ISG15 conjugates in *TRIM21*^*+/+*^ and *TRIM21*^*−/−*^ A549 cells, and found that all of them were induced by IFN-*β* without significant differences between these two cell lines in a time-dependent manner (Fig. 5C).

As the ISG15-modified substrates, including ISGylated TRIM21, were notably detected 24 h after IFN-*β* treatment (Fig. 5C), it is reasonable to test our hypothesis by detecting the K63-linked ubiquitination level of p62 at the same timepoint. To avoid interference of non-covalently interacting proteins in the ubiquitination assay, endogenous p62 protein was pulled down from IFN-*β*-treated A549 cells in denaturing RIPA buffer according to a previously reported protocol [35]. Our results revealed that K63-linked ubiquitination of p62 was significantly enhanced in *TRIM21*^*+/+*^, but not *TRIM21*^*−/−*^ A549 cells 24 h after IFN-*β* stimulation (Fig. 5D). Furthermore, it cannot be ruled out that the enhancement of K63-linked ubiquitination of p62 was caused by increased TRIM21 expression. To further assess the function of TRIM21 ISGylation in catalyzing K63-linked ubiquitination of p62, an empty vector and vector carrying wild-type TRIM21 or TRIM21(5KR) mutant were reintroduced into *TRIM21*^*−/−*^ A549 cells. These A549 cells were then stimulated with IFN-*β* for varying timepoints and harvested for endogenous denaturing immunoprecipitation assay. The results demonstrated that the K63-linked ubiquitination of p62 was obviously upregulated upon IFN-*β* stimulation only in *TRIM21*^*−/−*^ A549 cells stably expressing wild-type TRIM21, but not in the empty vector or TRIM21(5KR) mutant (Fig. 5E). Additionally, in *TRIM21*^*−/−*^ A549 cells stably expressing TRIM21(K279R) mutant, which blocked most multi-ISGylation (top band) on TRIM21 (Fig. 3D), the upregulation of p62 K63-linked ubiquitination was evidently abolished as compared to *TRIM21*^*−/−*^ A549 cells stably expressing wild-type TRIM21 (Fig. S5). Taken together, our data suggest that ISGylated TRIM21 indeed promotes the K63-linked ubiquitination level of p62 due to its enhanced K63-linkage-specific E3 ligase activity.

It is well-known that K63-linked ubiquitination at the Lys7 site of p62 mediated by TRIM21 blocks the self-oligomerization of p62 [23], which is a prerequisite for its localization to autophagosome [22] and subsequent autophagic degradation. It was facile to explain the decreased protein level of p62 after IFN-*β* stimulation for different periods in *TRIM21*^*−/−*^ A549 cells compared to *TRIM21*^*+/+*^ A549 cells (Fig. 5D). When the autophagic degradation of p62 was inhibited in the presence of bafilomycin A1 (Baf-A1), which blocked the fusion of autophagosomes and lysosomes, the protein levels of p62 in *TRIM21*^*+/+*^ and *TRIM21*^*−/−*^ A549 cells both accumulated in a time-dependent manner upon IFN-*β* stimulation (Fig. S6). Taken together, the synthesis and degradation of p62 were maintained in balance in Baf-A1-untreated *TRIM21*^*+/+*^ A549 cells at early stage of IFN-*β* stimulation. Conversely, in Baf-A1-untreated *TRIM21*^*−/−*^ A549 cells, the degradation of p62 was faster than synthesis, due to the loss of function of TRIM21 ISGylation induced by IFN-*β* in inhibiting p62 autophagosome targeting and autophagic degradation. However, surprisingly, the protein levels of p62 were unexpectedly and markedly increased 24 h after stimulation with IFN-*β* in whole cell lysates of both *TRIM21*^*+/+*^ and *TRIM21*^*−/−*^ A549 cells with or without Baf-A1 treatment (Figs. 5D and S6). qRT-PCR analysis showed that mRNA levels of p62 transcripts were also markedly enhanced 24 h after IFN-*β* treatment (Fig. 5F). Similar results were also detected in the *TRIM21*^*−/−*^ A549 cells stably expressing the empty vector, wild-type TRIM21, and TRIM21(5KR) mutant (Fig. 5E, G). In summary, the expression of p62 was upregulated by IFN-*β* treatment at a later stage for unidentified reasons, consistent with the observation in Raw264.7 cells stimulated by IFN-*γ* [36].

### p62 is sequestered from autophagosomes by ISGylated TRIM21 in IFN-*β* induced autophagy

As mentioned above, autophagy, as well as ISGylation, was found to be induced by type I IFNs recently [3, 26–28]. However, the relationship between them remains to be fully investigated. p62, a well-known autophagy selective receptor, was demonstrated to be modified by ISGylated TRIM21-catalyzed K63-linked ubiquitination (Fig. 5D, E), which has been shown to block its self-oligomerization [23]. Since self-oligomerization is required for p62 targeting to the autophagosome formation site [22], p62 should be sequestered from the autophagosome by ISGylated TRIM21 upon stimulation by IFN-*β*.

**Fig. 6:**
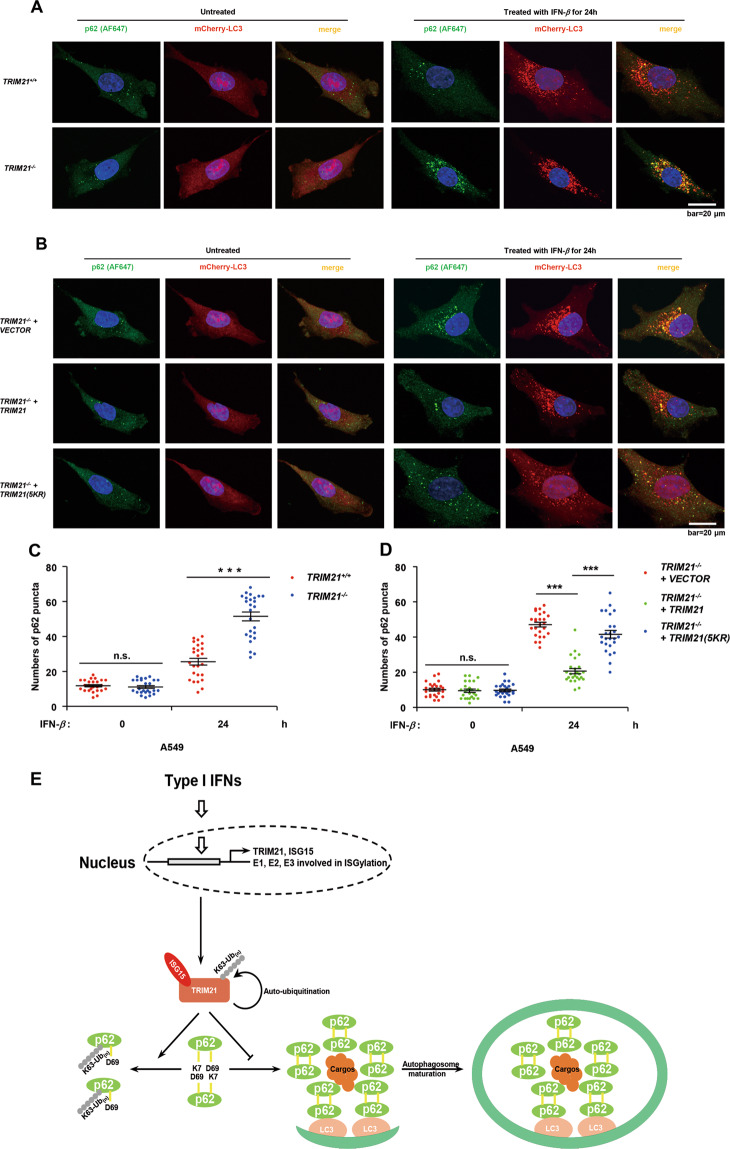
IFN-β-induced TRIM21 ISGylation prevents p62 oligomerization and autophagosome targeting. **A** *TRIM21*^*+/+*^ and *TRIM21*^*−/−*^ A549 cells were untreated or treated with IFN-*β* for 24 h. Cells were fixed, permeabilized, and then subjected to staining with anti-p62 antibody (green). Co-localization of p62 puncta and mCherry-LC3 labeled autophagosomes (red) were observed under Andor Dragonfly turntable confocal microscope. **B** *TRIM21*^*−/−*^ A549 cells stably expressing VECTOR, TRIM21, or TRIM21(5KR) mutant were untreated or treated with IFN-*β* for 24 h. Cells were subjected to staining with anti-p62 antibody and observed under confocal microscope as **A**. **C**, **D** p62 puncta in *TRIM21*^*+/+*^ and *TRIM21*^*−/−*^ A549 cells (**C**) or *TRIM21*^*−/−*^ A549 cells stably expressing VECTOR, TRIM21, or TRIM21(5KR) mutant (**D**) untreated or treated with IFN-*β* for 24 h were quantified with Imaris. Data shown were the statistical result of 25 cells for each cell line in 28 independent experiments plotted with GraphPad Prism 5. Data were analyzed with Student’s *t* test and represented as mean ± standard error of the mean (n.s. not significant. ***P < 0.001). **E** A working model for regulation of p62 self-oligomerization and autophagosome targeting by TRIM21 ISGylation. Induction of TRIM21 ISGylation by IFN-*β* activates TRIM21 to catalyze K63-linked ubiquitination of itself and p62, which results in blocking of p62 self-oligomerization and subsequent localizing to autophagosome formation site.

To verify our results, we constructed mCherry-LC3 overexpressing A549 cell lines with lentiviruses and performed immunofluorescence staining and imaging experiments with p62 antibody to examine the co-localization of p62 puncta and mCherry-LC3-labeled autophagosomes. Compared to *TRIM21*^*+/+*^ A549 cells, IFN-*β* treatment led to significant increases in the numbers of co-located puncta of p62 and autophagosomes in *TRIM21*^*−/−*^ A549 cells (Figs. 6A, C and S7C). Consistent with these findings, reconstitution of wild-type TRIM21, but not the TRIM21(5KR) mutant, led to diminished co-location of the puncta of p62 and autophagosomes (Figs. 6B, D and S7D). Together, these results demonstrate that ISGylation of TRIM21 induced by IFN-*β* plays a vital role in p62 oligomerization and autophagosome targeting by catalyzing its K63-linked ubiquitination.

## Discussion

In this study, we demonstrated that the E3 ubiquitin ligase TRIM21 is a substrate of ISGylation, which is catalyzed by E3 ISG15 ligase HERC5 and deISGylated by USP18. Surprisingly, the ISGylated TRIM21 bands were detectable, irrespective of the treatment of A549 cells with IFN-*β*. The difference is that only the top band, but not the bottom band, was inducible by IFN-*β* stimulation. This result indicates that mono-ISGylation of TRIM21 plays more universal and as yet unknown roles in unidentified physiological processes. In the HEK293 overexpression system, we identified two major ISGylation sites, Lys260 and Lys279, and a subset of alternative sites that were ISGylated when Lys260 and Lys279 were mutated to arginine, using immunoprecipitation coupled with LC-MS/MS analysis. Consistent with the results of recent studies [11, 30], mutations in the major ISGylation sites, Lys260 and Lys279, as well as neighboring lysine residues, Lys214, Lys217, and Lys280, blocked the formation of the top ISGylation band of TRIM21. Furthermore, mutation of these five lysine residues to arginine prevents the formation of the multi-ISGylated TRIM21(5KR) in the top band, resulting in the marked increase of mono-ISGylated TRIM21(5KR) in the low band.

Similar to the cases of CHIP [32] and parkin [33], we found that the E3 ubiquitin ligase activity of TRIM21 was also enhanced by ISGylation. However, unlike ISGylation of CHIP [32] and parkin [33], TRIM21 ISGylation enhanced its activity for K63-linked ubiquitination, but not for K48-linked ubiquitination. Furthermore, ISGylation of TRIM21 induced by IFN-*β* also led to enhanced K63-linkage-specific E3 ligase activity in A549 cells, which increased the K63-linked ubiquitination levels of TRIM21 and p62. It was recently reported that TRIM21 maintains its E3 ligase activity at a low level through B-Box domain-mediated auto-inhibition at resting state, and can be activated by phosphorylation at Ser80 upon stimulation [37]. Thus it is rational to hypothesize that ISGylation of TRIM21 induced by IFN-*β* stimulation has similar effects as that of Ser80 phosphorylation of TRIM21.

TRIM21-mediated K63-linked ubiquitination of p62 at Lys7 has been reported to inhibit its self-oligomerization [23], which is essential for its localization to autophagosome [22]. Our findings suggest that ISGylated TRIM21 blocks p62 self-oligomerization and subsequent packaging by autophagosomes in IFN-*β*-induced autophagy. Consistent with our findings, more and larger p62 puncta were formed and localized to autophagosomes 24 h after IFN-*β* treatment in *TRIM21*^*−/−*^ A549 cells than in the *TRIM21*^*+/+*^ A549 cells (Figs. 6A, C and S7C). Additionally, reconstitution of wild-type TRIM21, but not the TRIM21(5KR) mutant, into *TRIM21*^*−/−*^ A549 cells reduced the number and size of co-located p62 puncta in autophagosomes 24 h after IFN-*β* treatment (Figs. 6B, D and S7D). Nonetheless, there seemed to be no significant differences in the numbers of total LC3B puncta between IFN-*β*-treated *TRIM21*^*+/+*^ and *TRIM21*^*−/−*^ A549 cells (Fig. S7A) or *TRIM21*^*−/−*^ A549 cells stably expressing empty vector, wild-type TRIM21, and TRIM21(5KR) mutant (Fig. S7B).

It has recently been amply documented that TRIM21 functions as a cytosolic Fc receptor sensing incoming antibody-coated viruses and then inducing the expressing of type I IFNs through immune signaling as the first wave immune response [16]. With the help of AAA ATPase p97/VCP, TRIM21 mediates the proteasomal degradation of viral capsid and antibody by catalyzing autoubiquitination [38, 39]. In addition, TRIM21 catalyzes not only the K27-linked ubiquitination of MAVS [19] and K63-linked ubiquitination of Nmi [20] to regulate RIG-I signaling, but also the mono-ubiquitination of IKK*β* [18, 40] and K48-linked ubiquitination of IRF3 [41] to regulate the activation of transcription factors NF-*κ*B and IRF3. In facilitating the proteasomal degradation of viral capsid and antibody, TRIM21 itself is also degraded by proteasome. We propose that the upregulation of TRIM21 induced by type I IFNs may function as a complementary mechanism to maintain the antiviral responses.

In summary, the current study provides a novel regulatory mechanism of p62 oligomerization and autophagosome targeting in autophagy induced by IFN-*β* via TRIM21 ISGylation in human non-small-cell lung carcinoma A549 cells.

## Materials and methods

### Cell culture and transfection

HEK293 and A549 cells were cultured in DMEM (WISENT INC., 319-005-CL) and RPMI 1640 (WISENT INC., 350-600-CL) respectively containing 10% FBS (PAN-Biotech, P30-3302) and 1% streptomycin-penicillin (WISENT INC., 450-201-el) at 37 °C in 5% CO_2_. HEK293 and A549 cells used were purchased from the global bioresource center ATCC and kept in our laboratory. DNA constructs were transiently transfected into HEK293 cells with polyethylenimine (PEI) (Sigma-Aldrich, 9002-98-6) and A549 cells with Lipofectamine 3000 (Invitrogen, L3000-015).

### Co-immunoprecipitation and western blot assays

Cells were harvested from 10 cm dishes 24 h after transfection and lysed with 500 μl Western & IP lysis buffer (Beyotime, P0013) containing 1× PMSF (Solarbio, P0100), protease inhibitor cocktail (bimake, B14002) and phosphatase inhibitor cocktail (bimake, B15001). Suspended cell lysates (400 μl) was taken to mix with 1 μg of control IgG or specific antibody and protein A/G agarose (Thermo Scientific, 20422) in a new 1.5 ml tube for immunoprecipitation. The mixture was rotated for 4 h or overnight at 4 °C, followed by centrifugation at 4000 rpm for 3 min and washed for five times with Western & IP lysis buffer. The immunoprecipitates and the rest cell lysates (100 μl) were prepared for western blot analysis by boiling at 98 °C for 10 min in equal volume 2× sample loading buffer.

The samples were subjected to SDS-PAGE and then transferred to 0.45 μm nitrocellulose (NC) membrane (Merck millipore, HATF00010). NC membranes were blocked in 5% non-fat milk prepared with Tris-buffered salined with Tween 20 (TBST) for 1 h, and incubated with corresponding primary antibody, second HRP-linked IgG in 5% non-fat milk for 2 h each successively. After each incubation, NC membranes were washed five times with TBST for 5 min each. Target proteins were detected with ECL reagent (Bio-rad, 1705061) and photographed by ChemiDoc^TM^ XRS + SYSTEM (Bio-Rad, 1708265).

### RNA extraction and qRT-PCR

Total RNA of A549 cells was extracted with TRNzol Universal Reagent (TIANGEN, DP424), reverse transcribed by using 5× EasyQuick RT MasterMix (CWBIO, CW2020M), and amplified and detected by LightCycler^®^ (Roche) with 2× UltraSYBR Mixture (CWBIO, CW0957M) in a final reaction volume of 10 μl. qRT-PCR primers were designed using Primer3 website, and the primer sequences for p62 were 5′-CACCTGTCTGAGGGCTTCTC-3′ and 5′-CACACTCTCCCCAACGTTCT-3′. Data were normalized by actin mRNA levels using primers, 5′-GGACTTCGAGCAAGAGATGG-3′ and 5′-AGCACTGTGTTGGCGTACAG-3′.

### Statistical analysis

Statistical data acquired in qRT-PCR and cell imaging are analyzed with Two-way ANOVA and Student’s *t* test by using GraphPad Prism 5, and are presented as mean ± standard error of the mean (S.E.M). *P* < 0.05 were considered statistically significant.

### Ubiquitination assays

Ubiquitination assays were performed in the denaturing RIPA lysis buffer (50 mM Tris-Cl, pH 7.4, 150 mM NaCl, 5 mM EDTA, 1% Triton X-100, 1% sodium pyrophosphate, 0.1% SDS) (Beyotime, P0013K) and protease inhibitor cocktail (bimake, B14002), with appropriate antibody to avoid disturbance by the ubiquitination of non-covalently interacting proteins. Unlike Co-Immunoprecipitation, the immunoprecipitates were washed for three times with RIPA lysis buffer. The saved cell lysates and the immunoprecipitates were subjected to western blot analysis to detect the target protein and its ubiquitination.

### Generation of TRIM21 KO A549 cells

sgRNA targeting TRIM21 (sgTRIM21 F: 5′-caccgCATGTTGGCTAGCTGTCGAT-3′, sgTRIM21 R: 5′-aaacATCGACAGCTAGCCAACATGc-3′) were synthesized, annealed and cloned into the px458 vector, which containing CRISPR/Cas9 and GFP encoding sequences. The px458-sgTRIM21 vector was transiently transfected into A549 cells with Lipofectamine 3000 and single cells were sorted into 96 wells plates by flow cytometry 24 h later when GFP was already expressed in the cells. Once the sorted cells were expanded into 24-wells plates, half of the cells were subjected to genome DNA extraction with TIANamp Genomic DNA Kit (TIANGEN, DP304-02), and amplification by PCR with identification primers (iTRIM21 F: 5′-CAGCCAAACCCCCTAAAGGT-3′, iTRIM21 R: 5′-AGAGGTGGTCCTCTCCCATT-3′). The PCR products were identified by DNA sequencing to choose genome edited clones.

### Construction of stable cell lines with lentivirus

pLVX-IRES-ZsGreen1 empty vector or vectors encoding genes of interest, together with packaging system VSV-G, pRRE and RSV-Rev were co-transfected into HEK293 cells with PEI. The medium was replaced by fresh medium with 10% FBS and 1% streptomycin-penicillin six hours later. The supernatants were harvested and concentrated with PEG6000 48 h later to infect A549 cells in the presence of 10 μg/ml polybrene.

### LC-MS/MS analysis

Flag-TRIM21 along with ISG15-conjugating system overexpressed in HEK293 cells was immunoprecipitated with anti-Flag affinity gel (bimake, B23102) 24 h after transfection and subjected to SDS-PAGE separation and Coomassie Blue staining. Target ISGylated bands were taken for in-gel digestion with trypsin according to a standard protocol, followed by Liquid chromatography-tandem mass spectrometry (LC-MS/MS) analysis on Thermo Scientific^TM^ Orbitrap Fusion^TM^ coupled with EASY-nLC^TM^ 1200. The MS/MS spectra of modified peptides were acquired by searching the raw data with Proteome Discoverer Program (Version 1.4).

### Immunofluorescence staining and imaging

A549 cells seeded on glass bottom culture dishes (φ15mm, TC) (NEST, 801002) were fixed with 4% formaldehyde for 10 min. Next, the cells were permeabilized in 0.5% saponin for 10 min and blocked in 5% goat serum for 1 h. The glass bottoms were covered by anti-p62 primary rabbit antibody in 1% BSA and stained with Alexa Fluor 647 linked goat anti-rabbit secondary antibodies subsequently. The cells were washed for three times 5 min each with PBS between each steps. Imaging was performed on Andor Dragonfly turntable confocal microscope. p62 puncta in different cell lines untreated or treated with IFN-*β* were quantified with Imaris.

### Antibody and reagents

Mouse control IgG (CST, 3900 S), HRP-linked horse anti-mouse IgG (CST, 7076 S) (1:2000), HRP-linked goat anti-rabbit IgG (CST,7074 S)(1:2000), Goat anti-rabbit IgG/Alexa Fluor 647 (Bioss, bs-0295G-AF647), rabbit anti-HA (CST, 3724 S)(1:1000), rabbit anti-Flag (CST, 2368 S)(1:1000), mouse anti-Flag (EASYBIO, BE2004-100), mouse anti-Myc (CST, 2276 S)(1:1000), rabbit anti-TRIM21 (proteintech, 12108-1-AP)(1:1000), rabbit anti-UBE1L (proteintech, 15818-1-AP)(1:1000), rabbit anti-UBCH8 (proteintech, 17278-1-AP)(1:1000), rabbit anti-HERC5 (GeneTex, GTX130167)(1:1000), mouse anti-HHARI (Santa Cruz biotechnology, sc-390763)(1:100), rabbit anti-ISG15 (Santa Cruz biotechnology, sc-50366)(1:500), rabbit anti-actin (ABclonal, AC004)(1:2000), rabbit anti-p62 (CST, 5114 S)(1:1000), rabbit Anti-Ubiquitin, Lys63-Specific (Merk millipore, 05-1308)(1:1000), Bafilomycin A1 (Selleck, S1413), IFNA2 (Sino Biological, 13833-HNAS), and IFN-*β* (Sino Biological, 10704-HNAS) were purchased from the corresponding manufactures.

### Short hairpin RNAs

The short hairpin RNA (shRNA) lentiviral plasmids pLKO-shControl, pLKO-shHHARI, pLKO-shEFP, and pLKO-shHERC5 were purchased from Custom Glycerol-human shRNA library Facility, Tsinghua University. The appropriate lentiviral plasmid together with packaging vectors VSV-G, pRRE, and RSV-Rev were co-transfected into HEK293 cells to obtain lentiviruses. Packaged viruses were then used to infect A549 cells in the presence of polybrene (10 μg/ml) and then selected with puromacin (1 μg/ml) for 2 weeks. Control vector expressed shRNA targeting TurboGFP. The sequences of shRNAs were listed below. shHHARI: 5′-CCGGGCTACCTTGAACGAGATATTTCTCGAGAAATATCTCGTTCAAGGTAGCTTTTTG-3′; shEFP: 5′-CCGGCCGGAACAGTTAGTGGATTTACTCGAGTAAATCCACTAACTGTTCCGGTTTTTG-3′; shHERC5: 5′-CCGGATGGGCAACTTGGTCATAATTCTCGAGAATTATGACCAAGTTGCCCATTTTTTG-3′.

### DNA constructs

pEGFP-N1-UBE1L-Myc, pEGFP-N1-UBCH8-Myc, pCDNA3.1-HERC5-Myc, pCMV-HA-ISG15, pCMV-HA-ISG15(AA), pCMV-Myc-ISG15, pCMV-Myc-ISG15(AA), pLVX-TRIM21-Flag-IRES-ZsGreen1, pLVX-ISG-TRIM21-Flag-IRES-ZsGreen1, pLVX-TRIM21-Myc-IRES-ZsGreen1, pLVX-ISG-TRIM21-Myc-IRES-ZsGreen1, pLVX-EFP-Myc-IRES-ZsGreen1, pLVX-HHARI-Myc-IRES-ZsGreen1, pLVX-HA-ubiquitin(WT)-IRES-ZsGreen1, pLVX-HA-ubiquitin(K48R)-IRES-ZsGreen1, pLVX-HA-ubiquitin(K63R)-IRES-ZsGreen1, pLVX-HA-ubiquitin(AKR)-IRES-ZsGreen1, pLVX-HA-ubiquitin(K48O)-IRES-ZsGreen1, pLVX-HA-ubiquitin(K63O)-IRES-ZsGreen1, pLVX-p62-Flag-IRES-ZsGreen1, pLVX-mcherry-LC3, pLKO-puro-shControl, pLKO-puro-shHHARI, pLKO-puro-shEFP, pLKO-puro-shHERC5, and px458-sgTRIM21.

## Supplementary information

Supplementary Figures

## References

[1] McNab F, Mayer-Barber K, Sher A, Wack A, O’Garra A (2015). Type I interferons in infectious disease. Nat Rev Immunol.

[2] Haas AL, Ahrens P, Bright PM, Ankel H (1987). Interferon induces a 15-kilodalton protein exhibiting marked homology to ubiquitin. J Biol Chem.

[3] Han HG, Moon HW, Jeon YJ (2018). ISG15 in cancer: beyond ubiquitin-like protein. Cancer Lett.

[4] Yuan W, Krug RM (2001). Influenza B virus NS1 protein inhibits conjugation of the interferon (IFN)-induced ubiquitin-like ISG15 protein. EMBO J.

[5] Zhao C, Beaudenon SL, Kelley ML, Waddell MB, Yuan W, Schulman BA (2004). The UbcH8 ubiquitin E2 enzyme is also the E2 enzyme for ISG15, an IFN-alpha/beta-induced ubiquitin-like protein. Proc Natl Acad Sci USA.

[6] Wong JJ, Pung YF, Sze NS, Chin KC (2006). HERC5 is an IFN-induced HECT-type E3 protein ligase that mediates type I IFN-induced ISGylation of protein targets. Proc Natl Acad Sci USA.

[7] Dastur A, Beaudenon S, Kelley M, Krug RM, Huibregtse JM (2006). Herc5, an interferon-induced HECT E3 enzyme, is required for conjugation of ISG15 in human cells. J Biol Chem.

[8] Zou W, Zhang DE (2006). The interferon-inducible ubiquitin-protein isopeptide ligase (E3) EFP also functions as an ISG15 E3 ligase. J Biol Chem.

[9] Okumura F, Zou W, Zhang DE (2007). ISG15 modification of the eIF4E cognate 4EHP enhances cap structure-binding activity of 4EHP. Genes Dev.

[10] Malakhov MP, Malakhova OA, Kim KI, Ritchie KJ, Zhang DE (2002). UBP43 (USP18) specifically removes ISG15 from conjugated proteins. J Biol Chem.

[11] Xu D, Zhang T, Xiao J, Zhu K, Wei R, Wu Z (2015). Modification of BECN1 by ISG15 plays a crucial role in autophagy regulation by type I IFN/interferon. Autophagy.

[12] Zhao C, Denison C, Huibregtse JM, Gygi S, Krug RM (2005). Human ISG15 conjugation targets both IFN-induced and constitutively expressed proteins functioning in diverse cellular pathways. Proc Natl Acad Sci USA.

[13] Foss S, Watkinson R, Sandlie I, James LC, Andersen JT (2015). TRIM21: a cytosolic Fc receptor with broad antibody isotype specificity. Immunol Rev.

[14] Keeble AH, Khan Z, Forster A, James LC (2008). TRIM21 is an IgG receptor that is structurally, thermodynamically, and kinetically conserved. Proc Natl Acad Sci USA.

[15] Mallery DL, McEwan WA, Bidgood SR, Towers GJ, Johnson CM, James LC (2010). Antibodies mediate intracellular immunity through tripartite motif-containing 21 (TRIM21). Proc Natl Acad Sci USA.

[16] McEwan WA, Tam JC, Watkinson RE, Bidgood SR, Mallery DL, James LC (2013). Intracellular antibody-bound pathogens stimulate immune signaling via the Fc receptor TRIM21. Nat Immunol.

[17] Wada K, Kamitani T (2006). Autoantigen Ro52 is an E3 ubiquitin ligase. Biochem Biophys Res Commun.

[18] Wada K, Niida M, Tanaka M, Kamitani T (2009). Ro52-mediated monoubiquitination of IKKβ down-regulates NF-*κ*B signalling. J Biochem.

[19] Xue B, Li H, Guo M, Wang J, Xu Y, Zou X (2018). TRIM21 promotes innate immune response to RNA viral infection through Lys27-linked polyubiquitination of MAVS. J Virol.

[20] Das A, Dinh PX, Pattnaik AK (2015). Trim21 regulates Nmi-IFI35 complex-mediated inhibition of innate antiviral response. Virology.

[21] Lamark T, Svenning S, Johansen T (2017). Regulation of selective autophagy: the p62/SQSTM1 paradigm. Essays Biochem.

[22] Itakura E, Mizushima N (2011). p62 Targeting to the autophagosome formation site requires self-oligomerization but not LC3 binding. J Cell Biol.

[23] Pan JA, Sun Y, Jiang YP, Bott AJ, Jaber N, Dou Z (2016). TRIM21 ubiquitylates SQSTM1/p62 and suppresses protein sequestration to regulate redox homeostasis. Mol Cell.

[24] Deretic V, Saitoh T, Akira S (2013). Autophagy in infection, inflammation and immunity. Nat Rev Immunol.

[25] Valečka J, Almeida CR, Su B, Pierre P, Gatti E (2018). Autophagy and MHC-restricted antigen presentation. Mol Immunol.

[26] Ambjørn M, Ejlerskov P, Liu Y, Lees M, Jäättelä M, Issazadeh-Navikas S (2013). IFNB1/interferon-β-induced autophagy in MCF-7 breast cancer cells counteracts its proapoptotic function. Autophagy.

[27] Zhu S, Cao L, Yu Y, Yang L, Yang M, Liu K (2013). Inhibiting autophagy potentiates the anticancer activity of IFN1@/IFNα in chronic myeloid leukemia cells. Autophagy.

[28] Schmeisser H, Fey SB, Horowitz J, Fischer ER, Balinsky CA, Miyake K (2013). Type I interferons induce autophagy in certain human cancer cell lines. Autophagy.

[29] Schmeisser H, Bekisz J, Zoon KC (2014). New function of type I IFN: induction of autophagy. J Interferon Cytokine Res.

[30] Park JM, Yang SW, Yu KR, Ka SH, Lee SW, Seol JH (2014). Modification of PCNA by ISG15 plays a crucial role in termination of error-prone translesion DNA synthesis. Mol Cell.

[31] Fletcher AJ, Mallery DL, Watkinson RE, Dickson CF, James LC (2015). Sequential ubiquitination and deubiquitination enzymes synchronize the dual sensor and effector functions of TRIM21. Proc Natl Acad Sci USA.

[32] Yoo L, Yoon AR, Yun CO, Chung KC (2018). Covalent ISG15 conjugation to CHIP promotes its ubiquitin E3 ligase activity and inhibits lung cancer cell growth in response to type I interferon. Cell Death Dis.

[33] Im E, Yoo L, Hyun M, Shin WH, Chung KC (2016). Covalent ISG15 conjugation positively regulates the ubiquitin E3 ligase activity of parkin. Open Biol.

[34] Yang L, Jin L, Ke Y, Fan X, Zhang T, Zhang C (2018). E3 ligase Trim21 ubiquitylates and stabilizes keratin 17 to induce STAT3 activation in psoriasis. J. Invest Dermatol.

[35] Peng H, Yang J, Li G, You Q, Han W, Li T (2017). Ubiquitylation of p62/sequestosome1 activates its autophagy receptor function and controls selective autophagy upon ubiquitin stress. Cell Res.

[36] Kim JY, Ozato K (2009). The sequestosome 1/p62 attenuates cytokine gene expression in activated macrophages by inhibiting IFN regulatory factor 8 and TNF receptor-associated factor 6/NF-κB activity. J Immunol.

[37] Dickson C, Fletcher AJ, Vaysburd M, Yang JC, Mallery DL, Zeng J (2018). Intracellular antibody signalling is regulated by phosphorylation of the Fc receptor TRIM21. Elife.

[38] Hauler F, Mallery DL, McEwan WA, Bidgood SR, James LC (2012). AAA ATPase p97/VCP is essential for TRIM21-mediated virus neutralization. Proc Natl Acad Sci USA.

[39] Watkinson RE, McEwan WA, Tam JCH, Vaysburd M, James LC (2015). TRIM21 promotes cGAS and RIG-I sensing of viral genomes during infection by antibody-opsonized virus. PLoS Pathog.

[40] Niida M, Tanaka M, Kamitani T (2010). Downregulation of active IKK beta by Ro52-mediated autophagy. Mol Immunol.

[41] Higgs R, Gabhann JN, Larbi NB, Breen EP, Fitzgerald KA, Jefferies CA (2008). The E3 ubiquitin ligase Ro52 negatively regulates IFN-beta production post-pathogen recognition by polyubiquitin-mediated degradation of IRF3. J Immunol.

