# Supplementary material for: Induced TRIM21 ISGylation by IFN-β enhances p62 ubiquitination to prevent its autophagosome targeting
Source: Cell Death Dis. 2021 Jul 13;12(7):697. doi: 10.1038/s41419-021-03989-x (PMC8277845; doi:10.1038/s41419-021-03989-x)
Supplement: Supplementary file 1 — Supplementary Figures [file 41419_2021_3989_MOESM1_ESM.pdf]

Figure S1

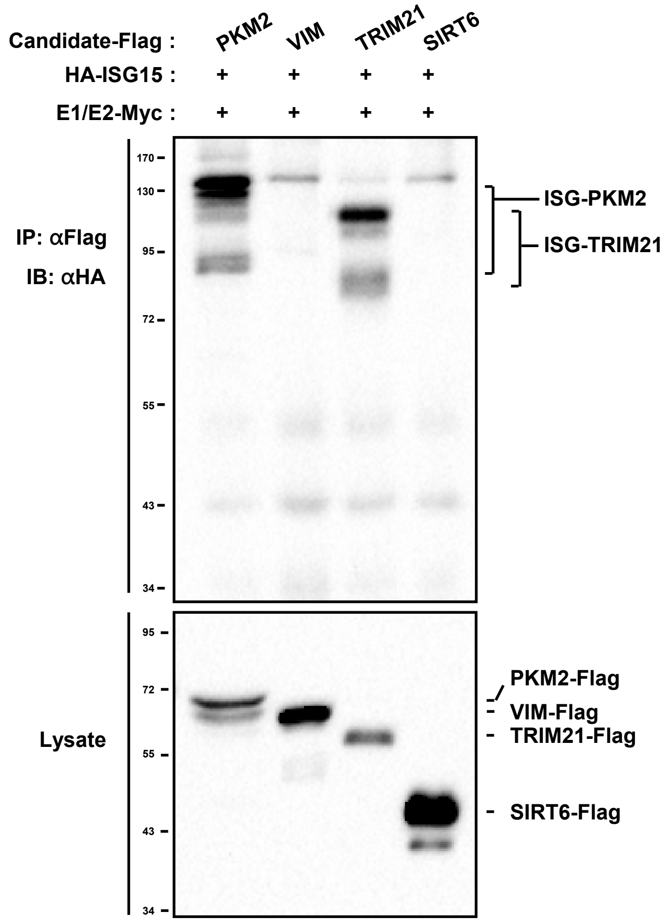

Fig.S1 Screening of candidate substrates of ISGylation. Flag-tagged candidate proteins such as PKM2, VIM, TRIM21, and SIRT6 were co-transfected with UBE1L-Myc (E1), UBCH8-Myc (E2), and HA-ISG15 into HEK293 cells. Cell lysates were subjected to immunoprecipitation with mouse Flag antibodies, followed by immunoblotting with HA antibodies to detect the ISGylated bands. Cell lysates were analyzed with HA antibodies. Data shown are representative of three independent experiments.

Figure S2

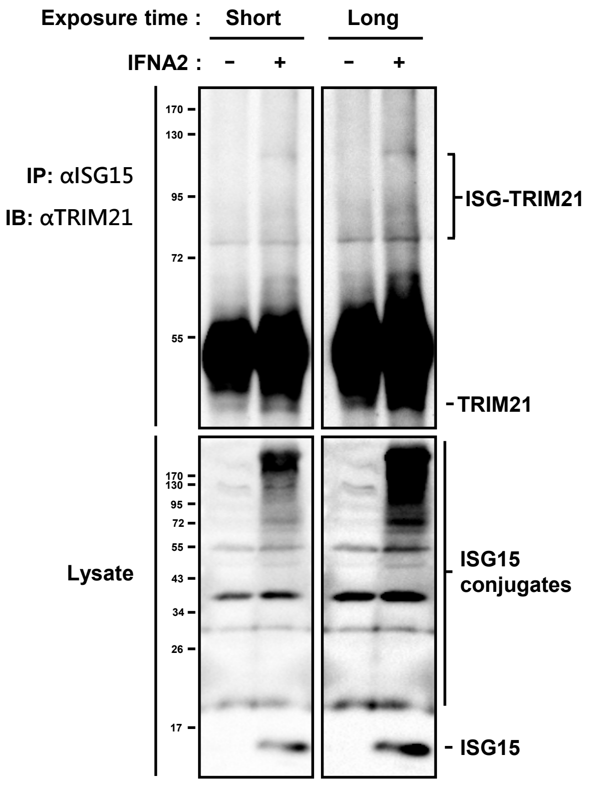

Fig.S2 TRIM21 ISGylation is also induced by IFNA2. A549 cells were treated with or without IFNA2 for 24 h. Cells were collected and lysed with RIPA buffer and subjected to immunoprecipitation with ISG15 antibodies, followed by immunoblotting with TRIM21 antibodies. Data shown are representative of three independent experiments.

Figure S3

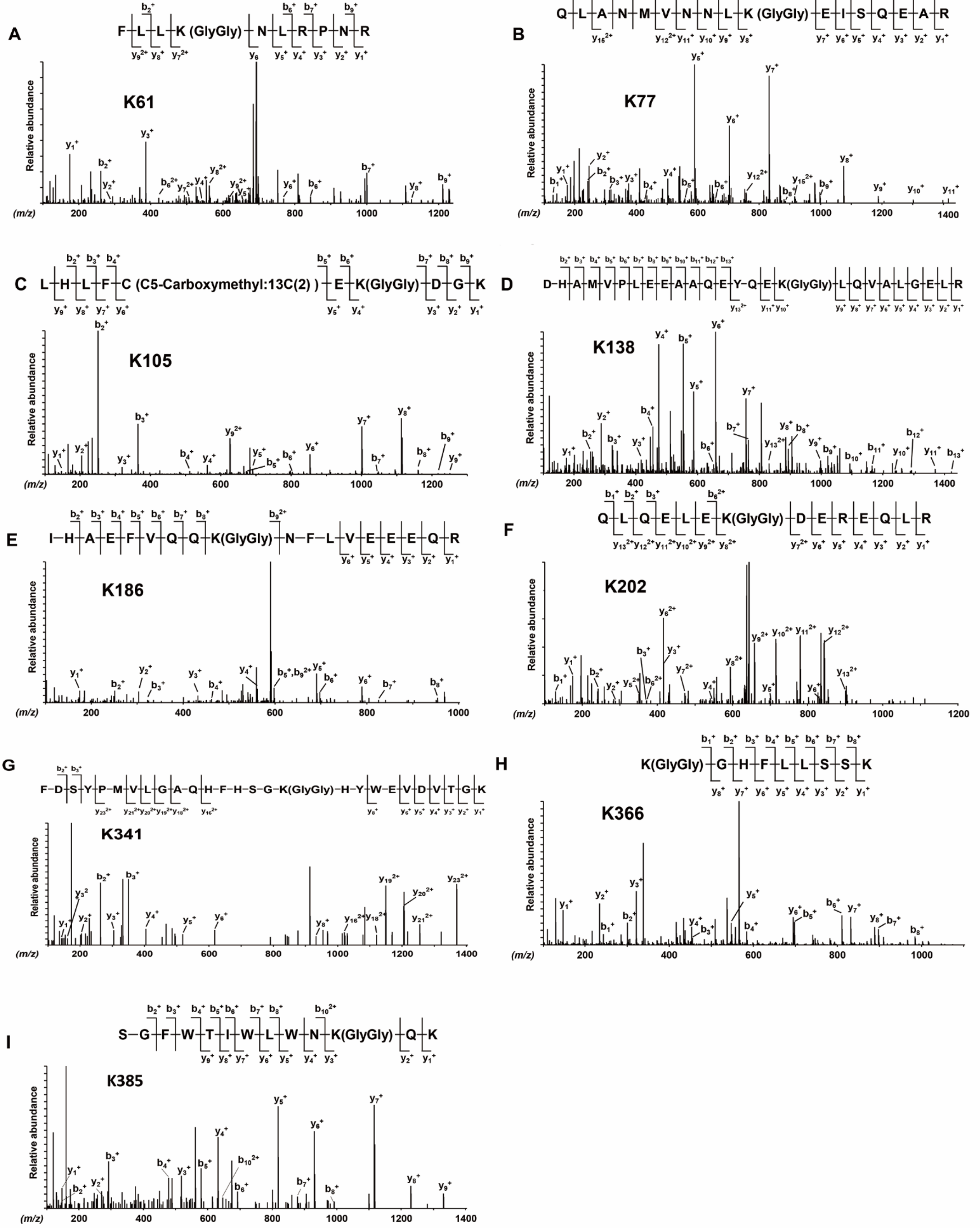

Fig.S3 Identification of ISGylation sites in TRIM21(5KR) mutant. A-I TRIM21-Flag (5KR) mutant were co-expressed with the ISG15-conjugating system in HEK293 cells. Cell lysates were pulled down with anti-Flag affinity gel, and then subjected to SDS-PAGE separation and in-gel digestion. Iodoacetic acid-$^{13}C_2$, instead of iodoacetamide, was used in in-gel digestion to avoid false positive result. LC-MS/MS analysis identified Lys61 (A), Lys77 (B), Lys105 (C), Lys138 (D), Lys186 (E), Lys202 (F), Lys341 (G), Lys366 (H), Lys 385 (I) as alternative ISGylation sites in the bottom ISGylated TRIM21 band.

Figure S4

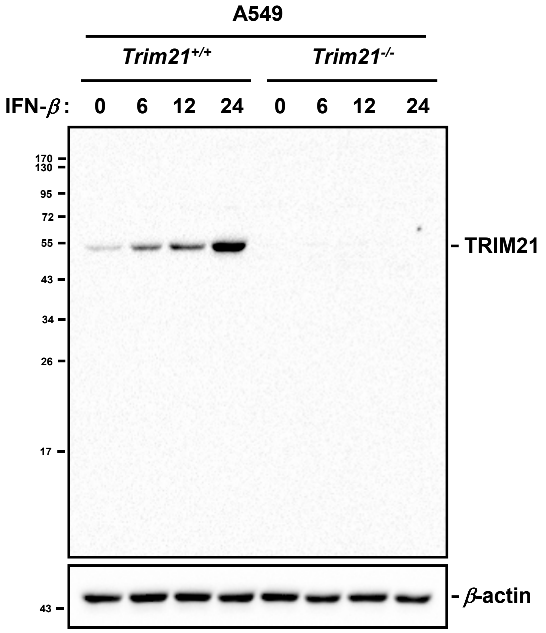

Fig.S4 Validation of TRIM21 KO cells generated using CRISPR/Cas9 technology. *TRIM21*$^{+/+}$ and *TRIM21*$^{-/-}$ A549 cells were treated with IFN-β for indicated timepoints. Cells were collected and lysed with RIPA buffer and subjected to immunoblotting with indicated antibodies. Data shown are representative of three independent experiments.

Figure S5

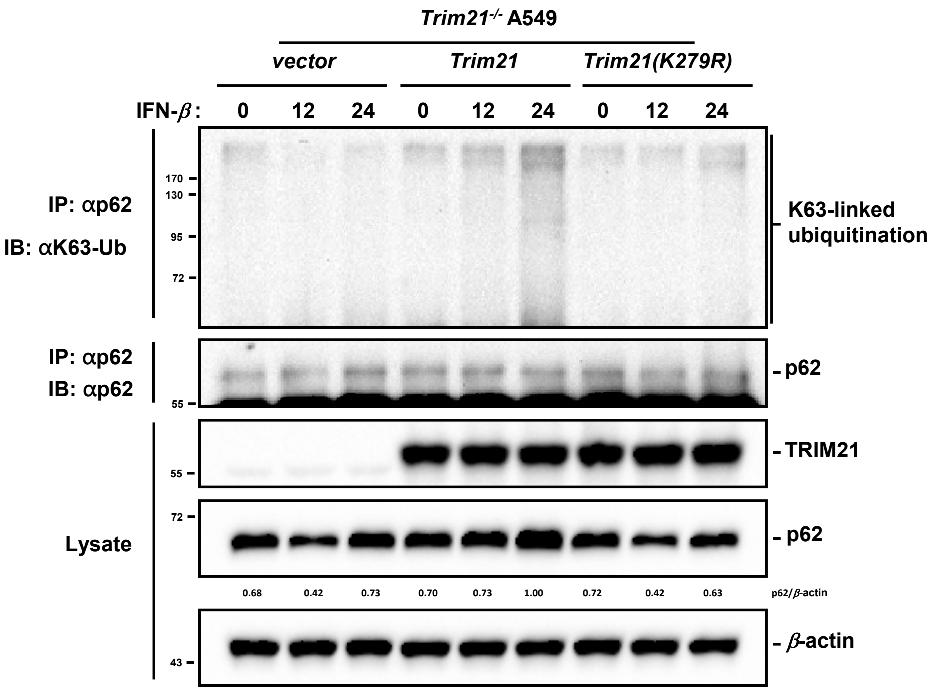

Fig.S5 TRIM21(K279R) mutant prevent K63-linked ubiquitination of p62 induced by IFN-*β*. Lysates of IFN-*β*-treated *TRIM21*$^{-/-}$ A549 cells stably expressing VECTOR, wild-type TRIM21, or TRIM21(K279R) mutant were immunoprecipitated in denaturing RIPA buffer with anti-p62 antibody at 4 °C overnight, followed by immunoblotting analysis to detect indicated proteins. Data shown are representative of three independent experiments.

Figure S6

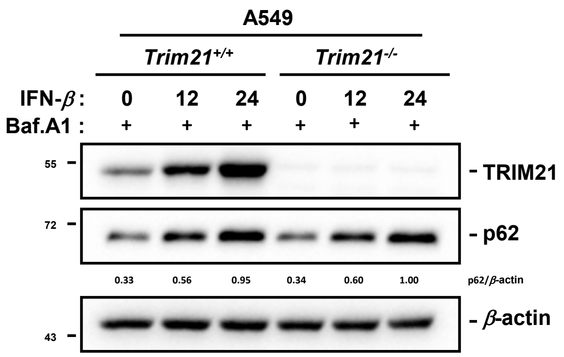

Fig.S6 Baf-A1 blocked the autophagic degradation of p62. A549 cells were treated with Baf-A1 (100 nM) for 24 h, and stimulated with IFN-β for indicated timepionts at the same time. Cells were collected and lysed with RIPA buffer and subjected to immunoblotting with indicated antibodies. Data shown are representative of three independent experiments.

Figure S7

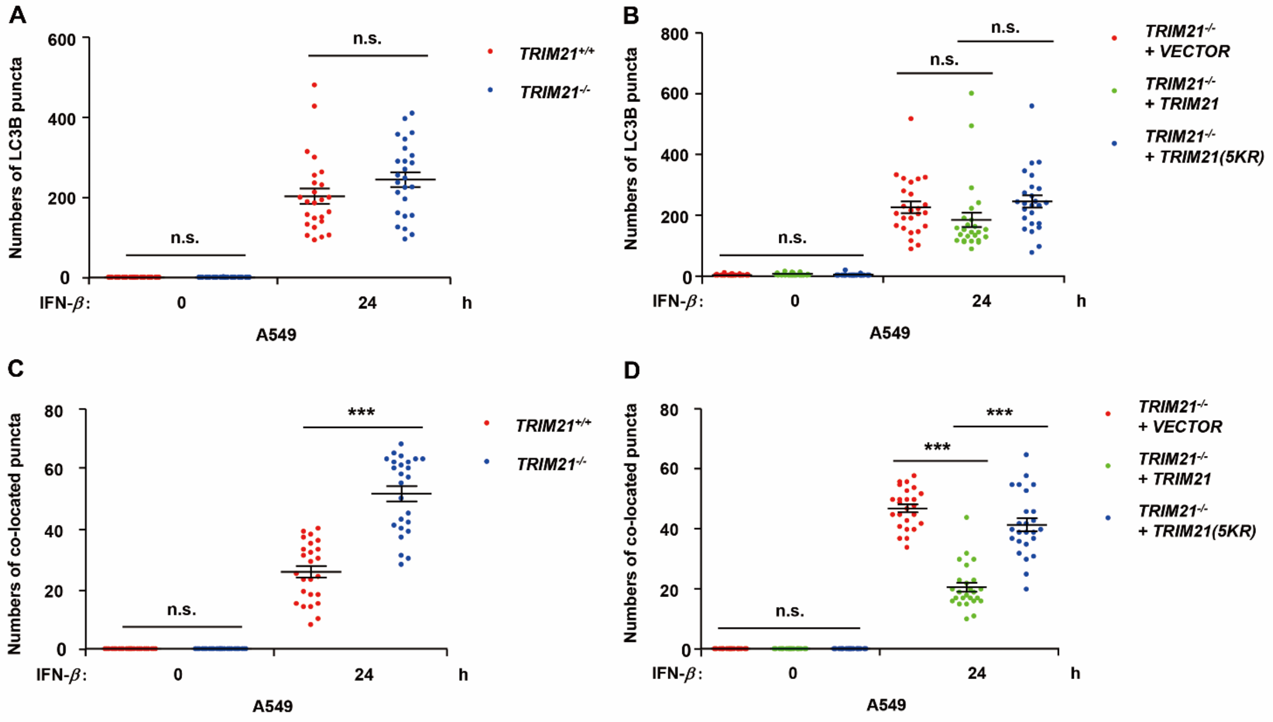

Fig.S7 Statistic analysis of total LC3B punta and co-localization of LC3 and p62 puncta. A-D Total LC3B puncta and co-located LC3 and p62 puncta in *TRIM21*$^{+/+}$ and *TRIM21*$^{-/-}$ A549 cells (A, C) or *TRIM21*$^{-/-}$ A549 cells stably expressing VECTOR, wild-type TRIM21, and TRIM21(5KR) mutant (B, D) untreated or treated with IFN-β for 24 h were quantified with Imaris. Data shown were the statistical result of 25 cells for each cell line in 28 independent experiments plotted with GraphPad Prism 5. Data were analyzed with Student's *t*-test and represented as mean ± standard error of the mean (n.s., not significant. ****P* < 0.001).
